# Gravity-driven controls on fluid and carbonate precipitation distributions in fractures

**DOI:** 10.1038/s41598-023-36406-8

**Published:** 2023-06-09

**Authors:** Zhenyu Xu, Hongfan Cao, Seonkyoo Yoon, Peter K. Kang, Young-Shin Jun, Timothy Kneafsey, Julia M. Sheets, David Cole, Laura J. Pyrak-Nolte

**Affiliations:** 1 Department of Physics & Astronomy, Purdue University, West Lafayette, IN 47907 USA; 2 Department of Earth and Environmental Sciences, University of Minnesota, Twin Cities, MN 55455 USA; 3 Department of Energy, Environmental and Chemical Engineering, Washington University in St. Louis, St. Louis, MO 63130 USA; 4 Lawrence Berkeley National Laboratory, Earth & Environmental Sciences, Berkeley, CA 94720 USA; 5 The Ohio State University, School of Earth Sciences, Columbus, OH 43210 USA; 6 Department of Earth, Atmospheric & Planetary Sciences, Purdue University, West Lafayette, IN 47907 USA; 7 Purdue University, Lyles School of Civil Engineering, West Lafayette, IN 47907 USA

**Keywords:** Hydrology, Geochemistry, Carbon capture and storage

## Abstract

Many challenges related to carbon-dioxide ($\hbox {CO}_2$) sequestration in subsurface rock are linked to the injection of fluids through induced or existing fracture networks and how these fluids are altered through geochemical interactions. Here, we demonstrate that fluid mixing and carbonate mineral distributions in fractures are controlled by gravity-driven chemical dynamics. Using optical imaging and numerical simulations, we show that a density contrast between two miscible fluids causes the formation of a low-density fluid runlet that increases in areal extent as the fracture inclination decreases from 90$^\circ$ (vertical fracture plane) to 30$^\circ$. The runlet is sustained over time and the stability of the runlet is controlled by the gravity-driven formation of 3D vortices that arise in a laminar flow regime. When homogeneous precipitation was induced, calcium carbonate covered the entire surface for horizontal fractures (0$^\circ$). However, for fracture inclinations greater than 10$^\circ$, the runlet formation limited the areal extent of the precipitation to less than 15% of the fracture surface. These insights suggest that the ability to sequester $\hbox {CO}_2$ through mineralization along fractures will depend on the fracture orientation relative to gravity, with horizontal fractures more likely to seal uniformly.

## Introduction

One method for reducing carbon-dioxide ($\hbox {CO}_2$) in the Earth’s atmosphere is to inject captured $\hbox {CO}_2$ into the Earth’s subsurface where there are several mechanisms that can trap or hold the $\hbox {CO}_2$ in place^1^. Subsurface $\hbox {CO}_2$ storage in rock through mineralization^2^ is tightly coupled to the properties of the injected and naturally occurring fluids, the reactivity and mineralogy along the fracture surfaces as well as by the morphology and connectivity of the fracture network through which the fluids flow. A field experiment in Iceland (Carbfix) showed that 95% of 220 tons of $\hbox {CO}_2$ injected into a subsurface basaltic reservoir in 2012 had been converted to calcite and other minerals^3^. In this process, $\hbox {CO}_2$ is dissolved into water (carbonic acid) and injected into a basaltic formation through a fracture network. The carbonic acid causes the release of cations from the basalt that in turn react with the carbonic solution to form carbonate minerals. These chemical processes not only alter the fracture surfaces but also affect the composition and density of the fluids, and in turn the hydrodynamics and fluid mixing within the fracture network.

This raises fundamental questions of how two miscible fluids with a density contrast mix and form mineral precipitates in a fracture. Mineral precipitation within a fracture is known to be affected by the flow path geometry within a fracture which controls the mixing^4^, by the diffusion and dispersion of fluids that control the extent and spatial distribution of fluid-rock interactions and mineralization^5^, and by mineral heterogeneity along the fracture flow paths that affect the type of induced mineral precipitation^4,6–10^. But a key factor not addressed in previous studies is the effect of fracture orientation relative to gravity on chemical dynamics. In horizontal fractures, fluid segregation occurs when the injected fluids have different densities, with the less dense fluid riding atop the denser fluid. For miscible fluids, a density gradient can lead to instabilities such as double diffusion-induced fingering^11^, convection driven mixing^12^ as well as Rayleigh-Taylor instabilities^13,14^. A key question is how these instabilities affect fluid mixing and in turn mineral precipitation across an inclined fracture plane.

In this paper, we combine visual laboratory experiments and numerical modeling to show that gravity-driven chemical dynamics control the mixing of fluids and precipitate distribution within a uniform aperture fracture. We demonstrate that a density contrast between the two fluids can lead to confinement of the less dense fluid to a narrow runlet. The size of the runlet depends on the orientation of the fracture plane relative to gravity. The runlet shape and stability are affected by gravity-induced 3D vortices in a laminar flow regime, and the vortices also affect the mixing lines and spatial distribution of carbonate precipitates across the fracture plane. The presence of gravity-induced instabilities in a laminar regime has the potential to affect the design and operation of subsurface operations in the sequestration of $\hbox {CO}_2$ by mineral trapping in fractured rock. Fractures in the subsurface may seal differently depending on the orientation thus affecting the ability of a fracture to self-heal especially if oriented vertically. Horizontal fractures are more likely to be uniformly sealed by mineral precipitation.

## Results

### Gravity driven fluid confinement in fractures

**Figure 1:**
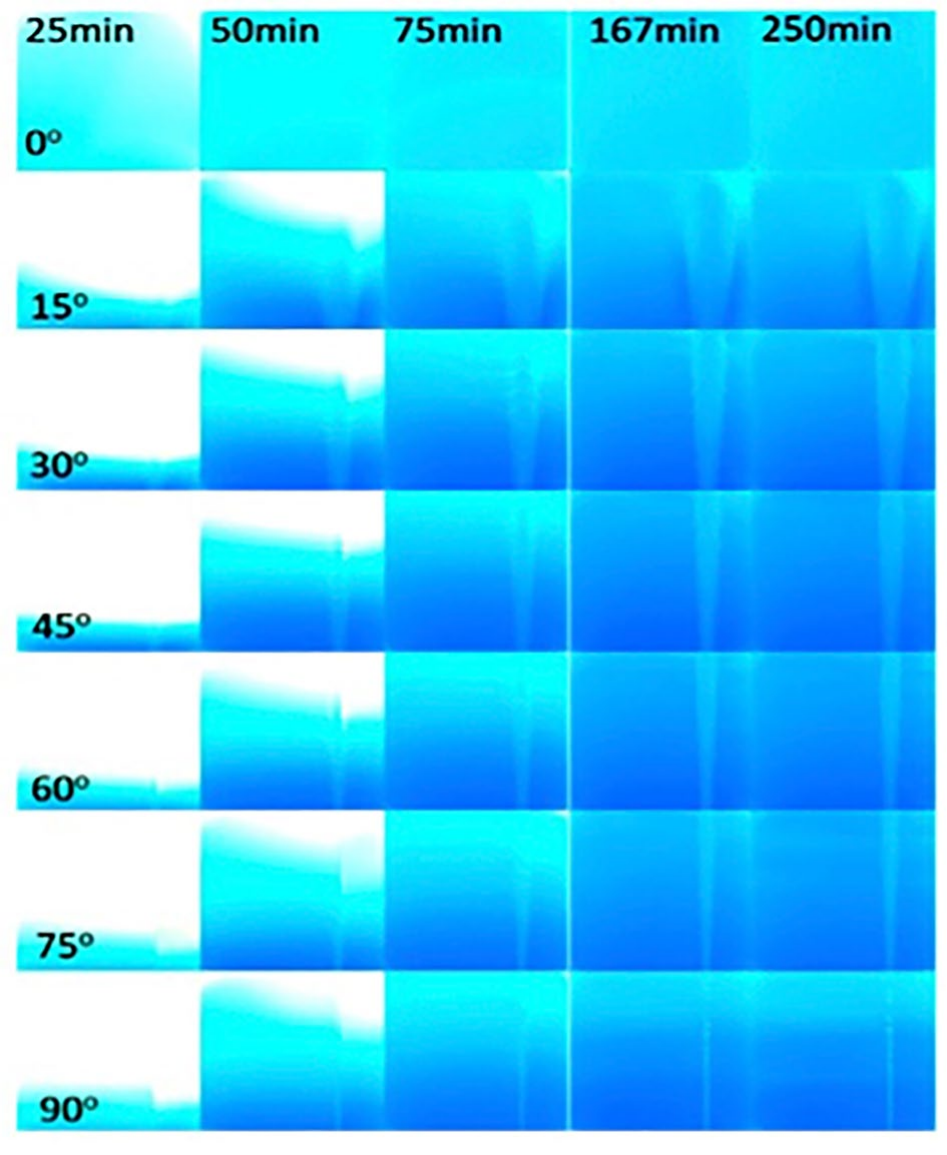
Enhanced digital images from non-reactive miscible fluid mixing experiments. Each column represents different times during the experiments (25, 50, 75, 167 and 250 minutes after the initiation of pumping of both fluids), and each row represents a different fracture inclination angle. The less dense Solution 2 was pumped in through the right port and Solution 1 through the left port. (see Supplement Information for port locations Figure S1.).

Experiments with non-reactive fluids were performed to understand how a density contrast alone affects the mixing of two fluids in inclined fractures (100 mm x 100 mm) with a uniform aperture of 2 mm (Figure S1 in Supplemental Information). The denser Solution 1 was composed of $\hbox {Na}_2$ $\hbox {CO}_3$, NaCl and water (see Table S1 in Supplemental Information) and introduced through the left inlet port. The less dense solution, Solution 2, was introduced through the right inlet port and consisted only of $\hbox {Na}_2$ $\hbox {CO}_3$ and water to yield a density contrast of approximately 7.1%. Solution 1 also contained Bromocresol green to enable imaging of the two solutions (Fig. 1). Initially, the fracture plane was saturated with Solution 2 and then both fluids were simultaneously pumped into the fracture plane at the same rate. A rate of 0.17 ml/min was used to ensure conditions were in the laminar flow regime (Reynolds number $\sim 1.34$). Solution 2 was pumped in through the right port (Figure S1) while the denser fluid (Solution 1), was pumped in through the left port at the same flow rate. (Note: Movies SM7 and SM8 of the fluid invasion for 0$^\circ$ and 90$^\circ$ can be found in the Supplemental Information.)

**Figure 2:**
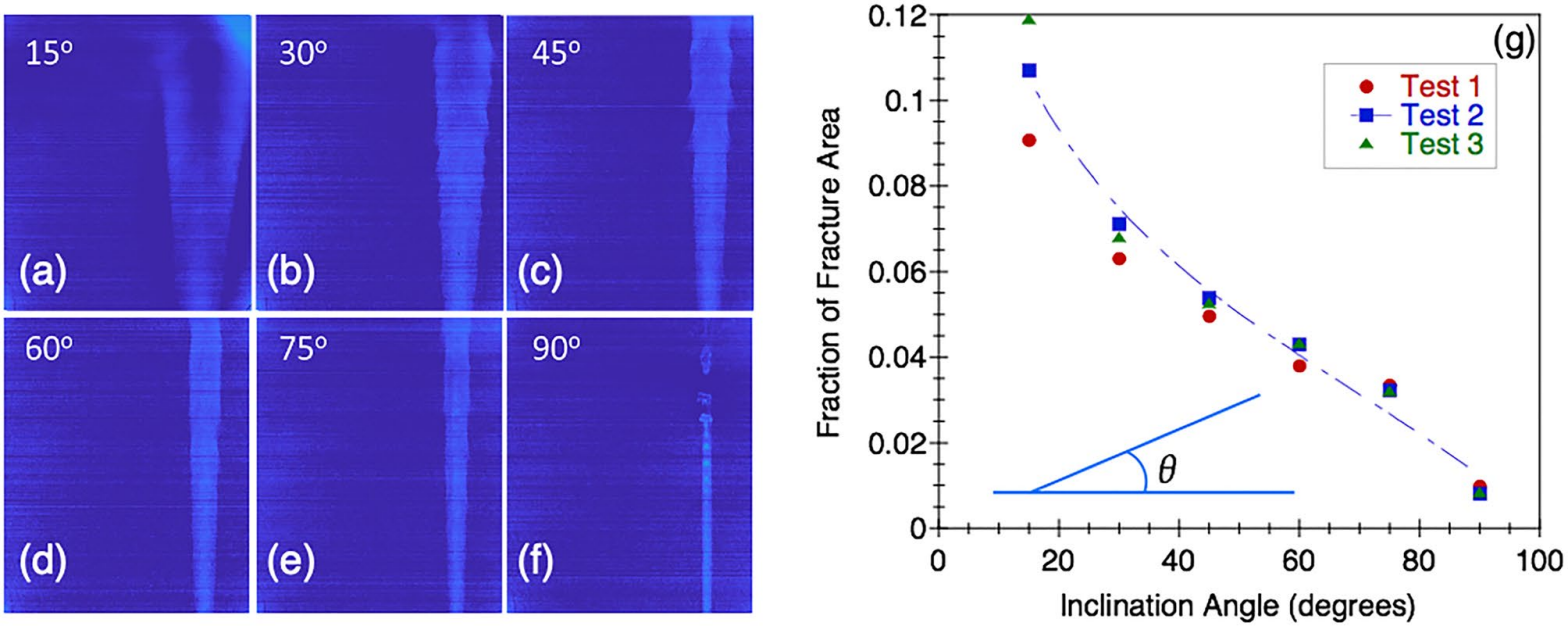
(**a**) False-color optical images of runlet geometry for (**a**–**f**) fracture inclinations of 0$^\circ$, 15$^\circ$ 30$^\circ$, 45$^\circ$, 60$^\circ$, 75$^\circ$ and 90$^\circ$ after 250 minutes. (**g**) Fraction of fracture area covered by the runlet as a function of fracture inclination (inset shows inclination angle).

Initially, the density contrast between the two fluids resulted in fluid stratification caused by gravity (at 25 min in Fig. 1) with the less dense fluid (white) on top of the denser fluid (blue). For the 90$^\circ$ fracture inclination, a narrow runlet of less dense fluid was observed to form directly over the inlet port of the less dense fluid. For fracture inclinations greater than 0$^\circ$, the runlet is observed to remain even after Solution 2 was completely displaced by the denser Solution 1 from the fracture plane (75 minutes). The width of the runlet increased with decreasing fracture inclination angles from 90$^\circ$ to 15$^\circ$ (Fig. 2). For inclination angles greater than 0$^\circ$, the runlet size stabilized after 167 minutes and did not change in spatial extent for the remaining 133 minutes of an experiment.

These experimental observations demonstrate that for fractures, the mixing and spatial distribution of miscible nonreactive fluids with different densities are affected by the relative orientation between the fracture plane and the gravity. The density contrast in a vertical fracture restricts the less dense fluid to a narrow path and causes hydrodynamic instabilities that create bubble-like features along the runlet (e.g. Fig. 1 90$^\circ$ at 250 minutes). For example, for an inclination angle of 90$^\circ$, discrete bubbles of the less dense solution are observed (Fig. 1 for times 50-250 minutes). While for fracture inclinations of 30$^\circ$ and 60$^\circ$, ripples along the perimeter of the runlet path are observed at 50 and 75 minutes (Fig. 2 on left). The runlet bifurcates into two branches near the outlet for fracture inclinations of 15$^\circ$ and 30$^\circ$.

Figure 2a–f provides a comparison of the runlet geometry at 250 minutes after the initiation of the simultaneous fluid invasion for the different fracture inclinations. To enhance the less dense solution in the images to estimate the area, a MATLAB-based code was used to subtract the background gradient of the fluid density concentration. The area of the less dense solution region was evaluated from the processed image for the different inclination angles and then normalized by the area of the fracture plane to give the fraction of fracture area value shown in Fig. 2g. As the fracture inclination angle decreased, the area of the less dense runlet increased. The change in area with angle of inclination is captured by csc($\theta$), which is related to the component of gravity parallel to the fracture plane.

**Figure 3:**
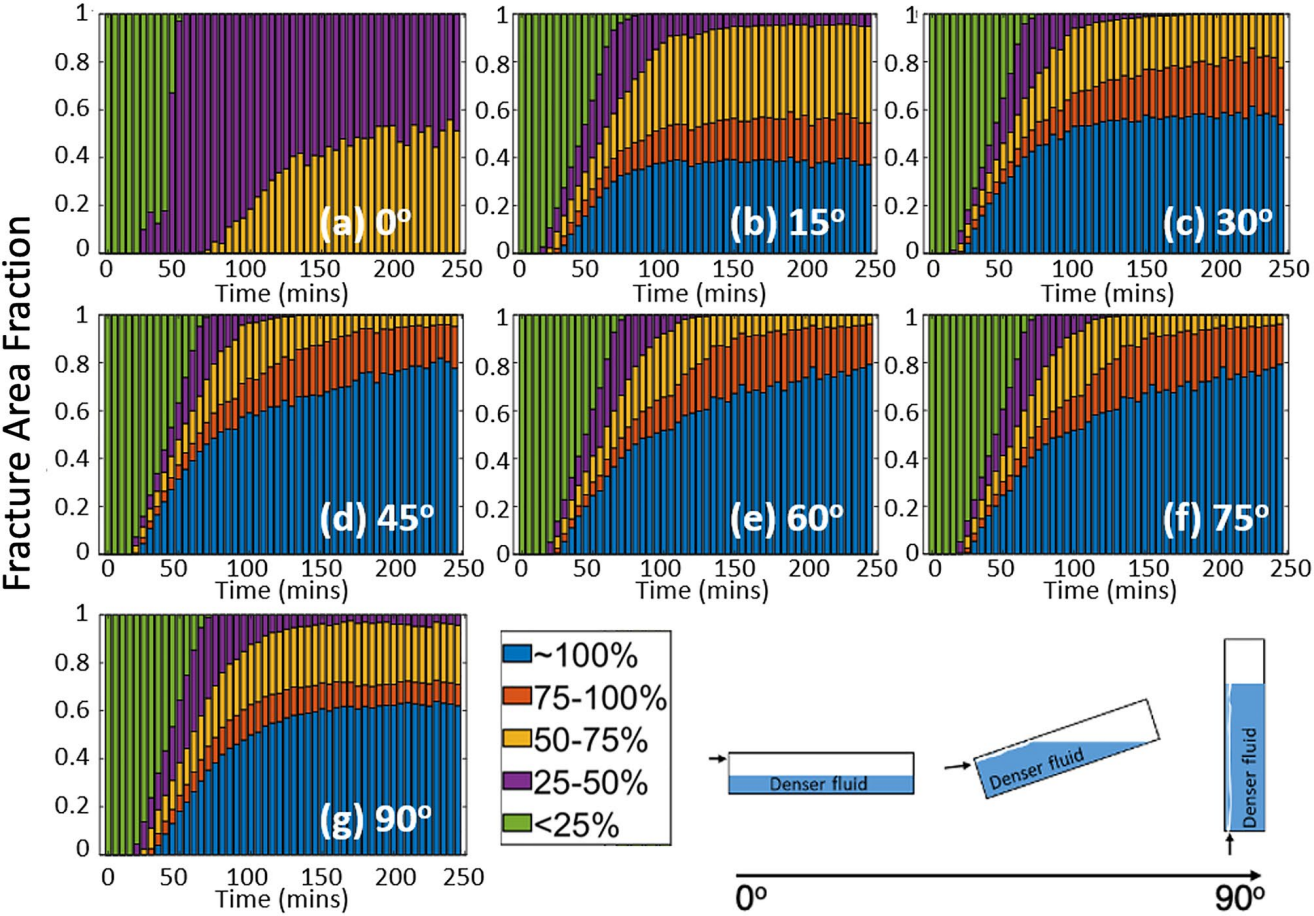
(**a**–**g**) Concentration of the denser fluid (Solution 1) as a function of experimental time. Inset is a sketch of fluid segregation as a function of fracture inclination angle.

Runlet formation was observed to affect fluid mixing across the fracture plane and in turn fluid concentrations. Figure 3 shows the time evolution of the concentration of the dense fluid (Solution1) for the different fracture inclinations (see Supplemental Information Section 5 for details). The light intensity gives information about projected average concentration and cannot distinguish the actual level of mixing. However, the 2D project concentration is sufficient to understand the effects of fracture orientation on spreading patterns. Also, mixing can be better understood from our experiments with precipitation and 3D numerical simulations presented in the next sections. The concentration of Solution 1 never reaches 100% (blue regions) in the horizontal fracture (0$^\circ$) contrary to the other fracture inclination angles. At 0$^\circ$, the fluids segregate perpendicular (i.e. in the 2 mm aperture) to the fracture plane (inset in Fig. 3) with the less dense fluid riding atop the denser fluid. For high inclination angles (45$^\circ$ to 75$^\circ$) by the end of the experiment, almost 80% of the fracture plane contains only the high-density fluid (100% Solution 1 blue regions). Because of the geometry of the gravity-induced runlet, the mixing between fluids occurs around the perimeter of the runlet and at the invading interface between Solutions 1 & 2 for times $< 80$ min. in inclined fractures, i.e., at the interface between the two fluids. The spatial extent of mixing increases as the inclination angle decreases. For fracture inclinations of $0^\circ$–$30^\circ$ and $90^\circ$, the fluid concentrations have reached steady-state. However, for inclinations from $45^\circ$–$75^\circ$, the concentrations are still changing in time which suggest both fluid segregation (Fig. 3) and runlet formation processes contribute to the fluid mixing.

Bubbles and ripples are observed in the runlets for fracture inclinations from 15$^\circ$–90$^\circ$ (Fig. 1). Instabilities can arise from both velocity and density differences between two fluids. In our experiments, a velocity contrast arises between the two fluids, though the fluids are pumped in at the same rate, because formation of a runlet reduces the area through which Solution 2 flows. Kelvin-Helmholtz (KH) instabilities occur along the interface between two fluids with different velocities^15^. Studies using horizontal Hele-Shaw cells found that the wavelength of KH instabilities between two immiscible fluids with different densities and viscosities was affected by the aperture of the cell. For a fixed aperture, the wavelength of the interface between gas and oil increased with distance from the inlet^16^. Rayleigh-Taylor (RT) instabilities can also occur at the interface between two fluids when the densities differ^13^. In studies of RT instabilities in a vertical Hele-Shaw cell^17,18^, the wavelength, $\lambda$, of the instabilities along the interface between the two fluids was observed to depend on the aperture, *b*, specifically $\lambda \sim 2b/3$ when diffusion is negligible (i.e. large Peclet Number, $Pe = b3\delta \rho g/\mu D$ where $\mu$ is the viscosity, $\delta \rho$ is the density difference, g is the acceleration caused by gravity). If the Pe is small, then $\lambda = b/Pe$.

**Figure 4:**
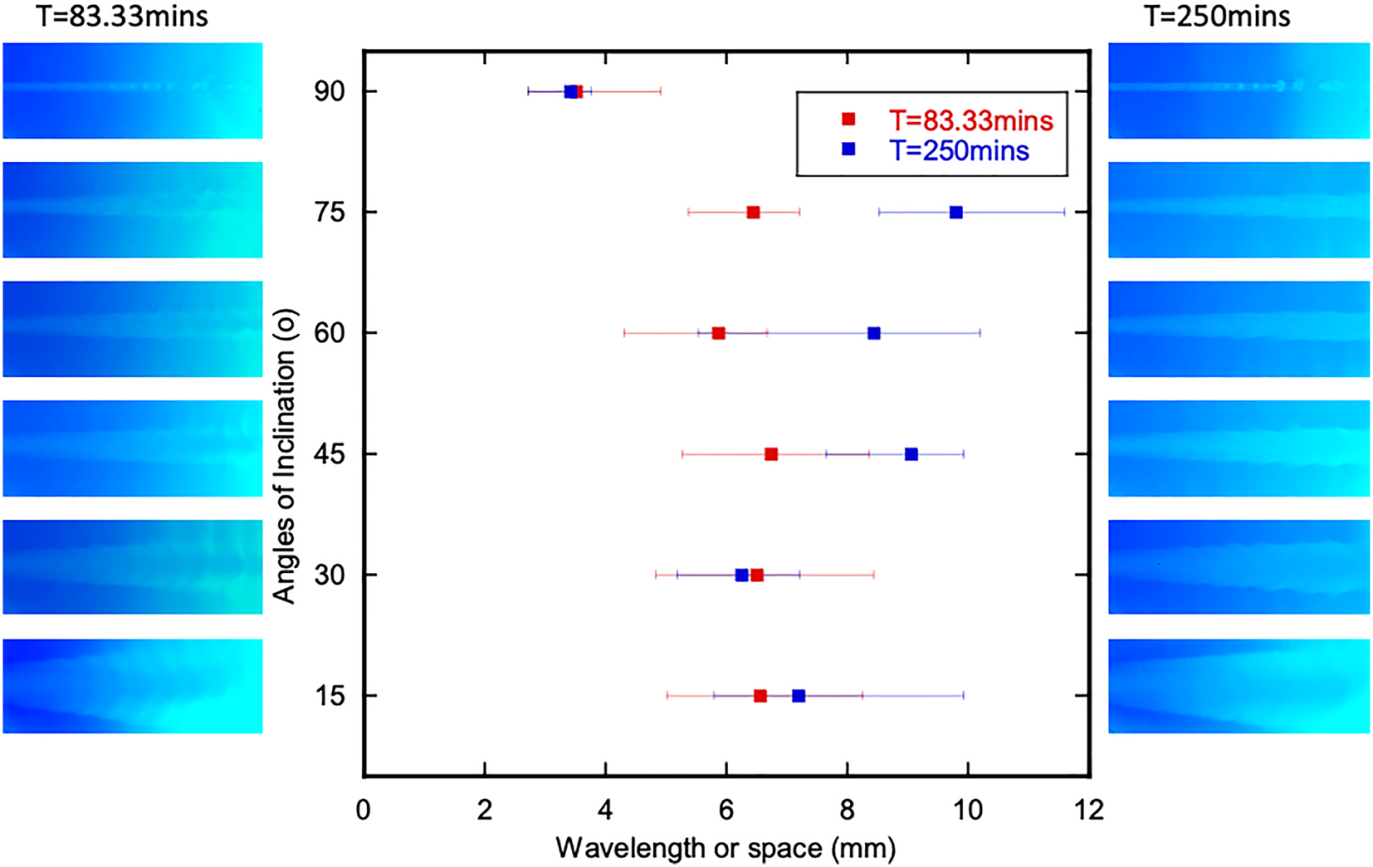
Values of space between the ripples at angles 15$^\circ$ to 90$^\circ$ at T=83.33 minutes and T=250 minutes. Aperture is 2 mm; pumping rate is 0.17 ml/min for both Solution 1 and 2; density contrast is 1111/1031.8. The small square represents the mean, and the left and right boundaries represented the minimum and maximum respectively.

We measured the bubble spacing and/or ripple wavelength for two different times to determine if the feature spacing changed over time. Figure 4 shows the bubble/ripple spacing observed in the runlets for the 15$^\circ$ - 90$^\circ$ fracture inclinations for times of 83.33 and 250 minutes after the initiation of pumping. The spacing was obtained by measuring the distance between the successive bulges along the runlet in the images. The average spacing between the ripples/bubbles for fracture inclinations of 15$^\circ$ to 75$^\circ$ range from 5.87 to 6.74 mm and vary with distance from the inlet which is contrary to the observations for KH instabilities for immiscible fluids^16^. For the 90$^\circ$ case, the spacing between the bubble-like features is 3.51mm and is relatively constant between 83.33 min and 250 min. The mean bubble/ripple spacing increased for inclination angles of 45$^\circ$ to 75$^\circ$. The measured spacings are on the order of the expected wavelength, $\lambda$, from Rayleigh-Taylor (RT) instabilities. Hydrodynamic instabilities affect the geometry of the mixing line between the two fluids which is important when the two fluids are reactive and precipitates form, and feature spacing of the instabilities are affected by gravity in terms of fracture inclination. The spacing analysis indicates that the instabilities are likely caused by RT instabilities and not KH instabilities.

### Gravity driven fluid confinement in fractures

**Figure 5:**
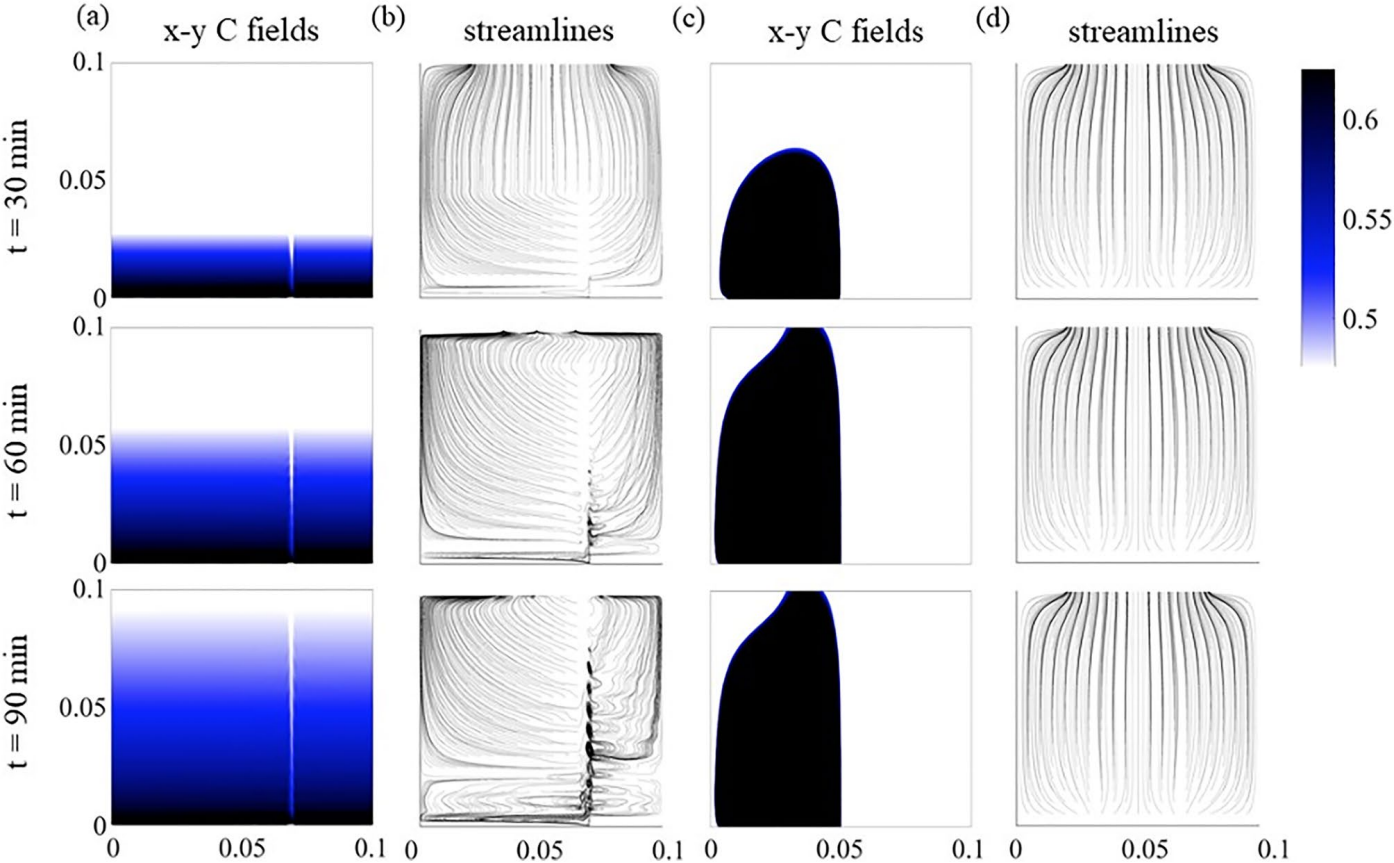
(**a**) Depth averaged concentration field in the x and y directions (i.e. fracture plane) of the case in which the two fluids have different density; (**b**) streamlines for the case in which the two fluids have different density; (**c**) depth averaged concentration field of the case in which the two fluids have same density; (**d**) streamlines for the case in which the two fluids have same density.

We conducted 3D pore-scale simulations to determine the mechanisms that result in the formation and instability of the runlets observed in the experiments. For a vertically inclined fracture, simulations were performed to examine how two fluids pumped simultaneously into a fracture lead to runlet formation. For comparison, the simulation was run first without a density contrast (Fig. 5c, d) and then with the same density contrast (Fig. 5a, b) used in the experiments. The governing equations used in the simulation and modeling assumptions can be found in the Supplemental Information Section 6 and Table S.5 lists the simulation parameters for the fluids for each case. The depth averaged concentration fields and streamlines are shown in Fig. 5. The depth averaged concentration fields were obtained by averaging concentration values in the aperture direction, and the streamlines were created based on the 3D velocity fields. By comparing the depth averaged concentration fields and streamlines, when there is a density difference between the two fluids, the lighter fluid is confined to a narrow path, i.e. a runlet, (Fig. 5a). Without a density difference, no runlet is formed (Fig. 5c) and the streamlines are dispersed (Fig. 5d). The fact that the horizontal fracture with density difference (Fig. 1 top row) and the vertical fracture without density difference (Fig. 5) do not cause the formation of a runlet confirms that both the density difference between the fluids and the fracture orientation with respect to the gravity are required for runlet formation.

From the streamlines (Fig. 5b), several vortices are observed to occur around the runlet. The overall runlet shape was maintained, but the runlet shape was unstable and showed small fluctuations with time. This phenomenon is similar to the Kelvin - Helmholtz - Rayleigh - Taylor instability which arises when the fluids with different densities have a relative velocity. The vortical flow structures near the runlet are similar to the convection rolls caused by the Rayleigh-Benard instability, which is a well-known phenomenon that is caused by a density gradient from a temperature difference. When a warmer fluid is located below a colder fluid, the density gradient caused by the temperature gradient induces a buoyancy force. The warmer, lighter fluid moves upwards, and the colder, heavier fluid sinks resulting in a flow pattern of convection rolls. The rotation of the rolls is usually stable, and a small perturbation will not affect the stability of the rolls. However, a larger perturbation can affect the rotation and trigger unstable flow^19,20^. In our system, although there is no temperature difference, there is density difference caused by the concentration difference between the fluids, which causes the lighter fluid to flow up and the denser fluid to flow down. Thus, the buoyancy force due to the concentration difference between the two fluids leads to Rayleigh - Taylor instability causing the unstable vortical flow structures near the runlet^13,14^. In addition to the buoyancy force, a relative velocity exists between the runlet flow and background flow. The runlet velocity is larger than the surrounding fluid because the injected fluid is focused through a narrow path (i.e. the runlet). The combination of the density contrast and the relative velocity breaks the stability of the convection rolls and leads to unstable vortical flow. This unstable vortical flow is the cause of the runlet instability and it affects the shape of the runlet through vortical flow motions. Further, the complex 3D streamlines around vortices strongly control mixing along the runlet, which in turn affect the runlet width and instability.

Through the 3D numerical simulations, we conclude that a density difference between fluids in an inclined fracture can lead to the runlet formation. The vortices are likely induced by a combination of the density contrast and the velocity difference between the fluids. The vortices are symmetric with respect to the runlet over time, thereby maintaining the overall runlet geometry.

### Gravity-controlled mineral distributions in inclined fractures

**Figure 6:**
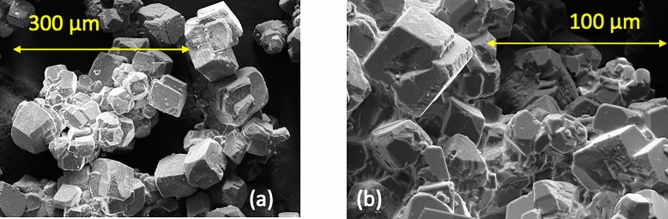
SEM image of (**a**) homogeneous and (**b**) heterogeneous calcium carbonate precipitates created using Eqs. (1) and (2–4) in a horizontal fracture.

The formation of runlets in inclined fractures is expected to affect mineralization along fractures because runlet formation limits the mixing lines between fluids and limits the areal extent that a fluid may interact with the rock. To test this hypothesis, calcium carbonate ($\hbox {CaCO}_3$) precipitation was induced in the uniform aperture (2mm) fractures (100 x 100 $\hbox {mm}^2$) for different fracture inclinations. Homogeneous precipitation was induced using Solution 3 (denser, introduced through left inlet port) with a 1 mol/L concentration calcium chloride ($\hbox {CaCl}_2$) in aqueous solution, and Solution 4 (less dense introduced through right inlet port) with a 0.3 mol/L concentration sodium carbonate ($\hbox {Na}_2\hbox {CO}_3$) in aqueous solution (see Table S.2 in Supplemental Information). These fluids produce the following reaction:

1 $$\begin{aligned} {CaCl_2 + Na_2CO_3 \rightarrow CaCO_3(s) + 2NaCl} \end{aligned}$$

that causes $\hbox {CaCO}_3$ to precipitate out of solution (Fig. 6a). As in the non-precipitation experiments, the fracture was initially filled with the less dense Solution 4 and then both solutions were simultaneously pumped into the fracture at a constant flow rate (0.17 ml/min) for 5 hours. Digital images were acquired every 5 seconds.

**Figure 7:**
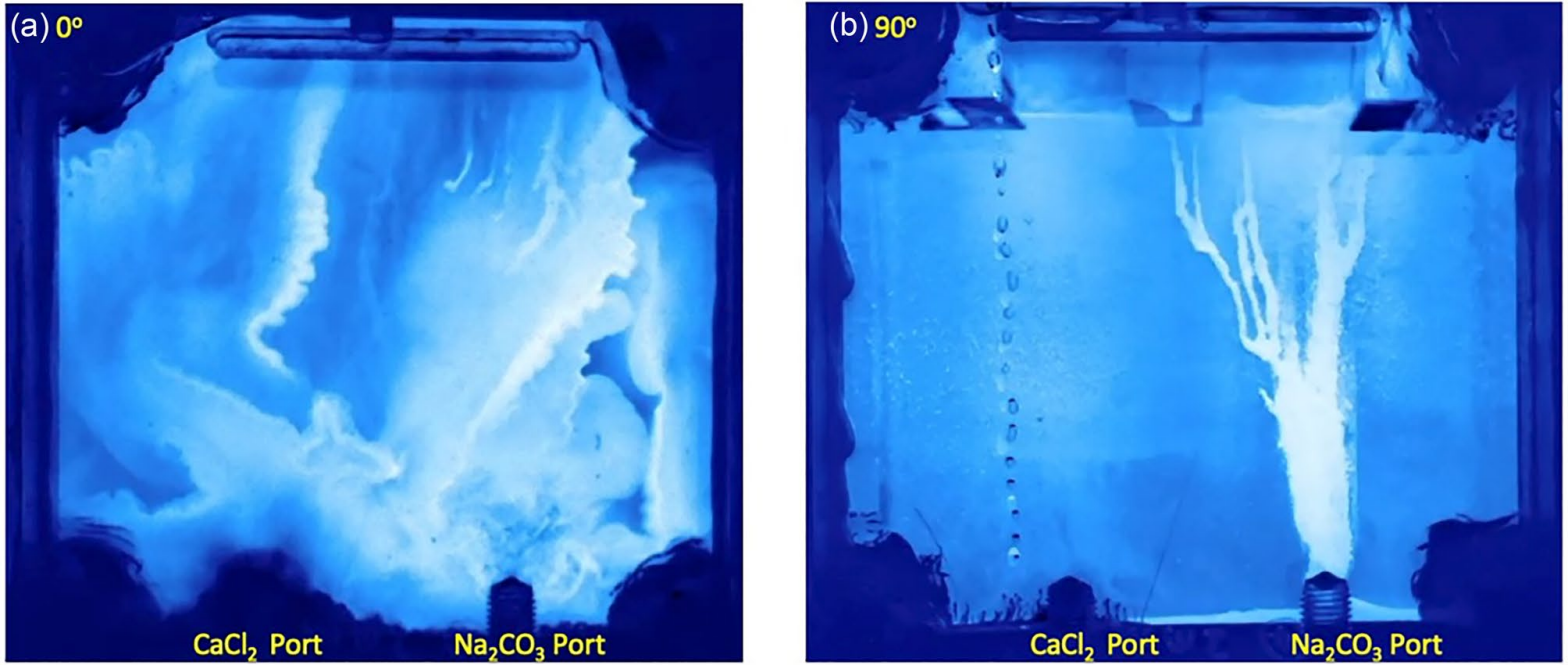
Precipitated calcite distributions in fractures inclined at (**a**) 0$^\circ$ and (**b**) 90$^\circ$.

**Figure 8:**
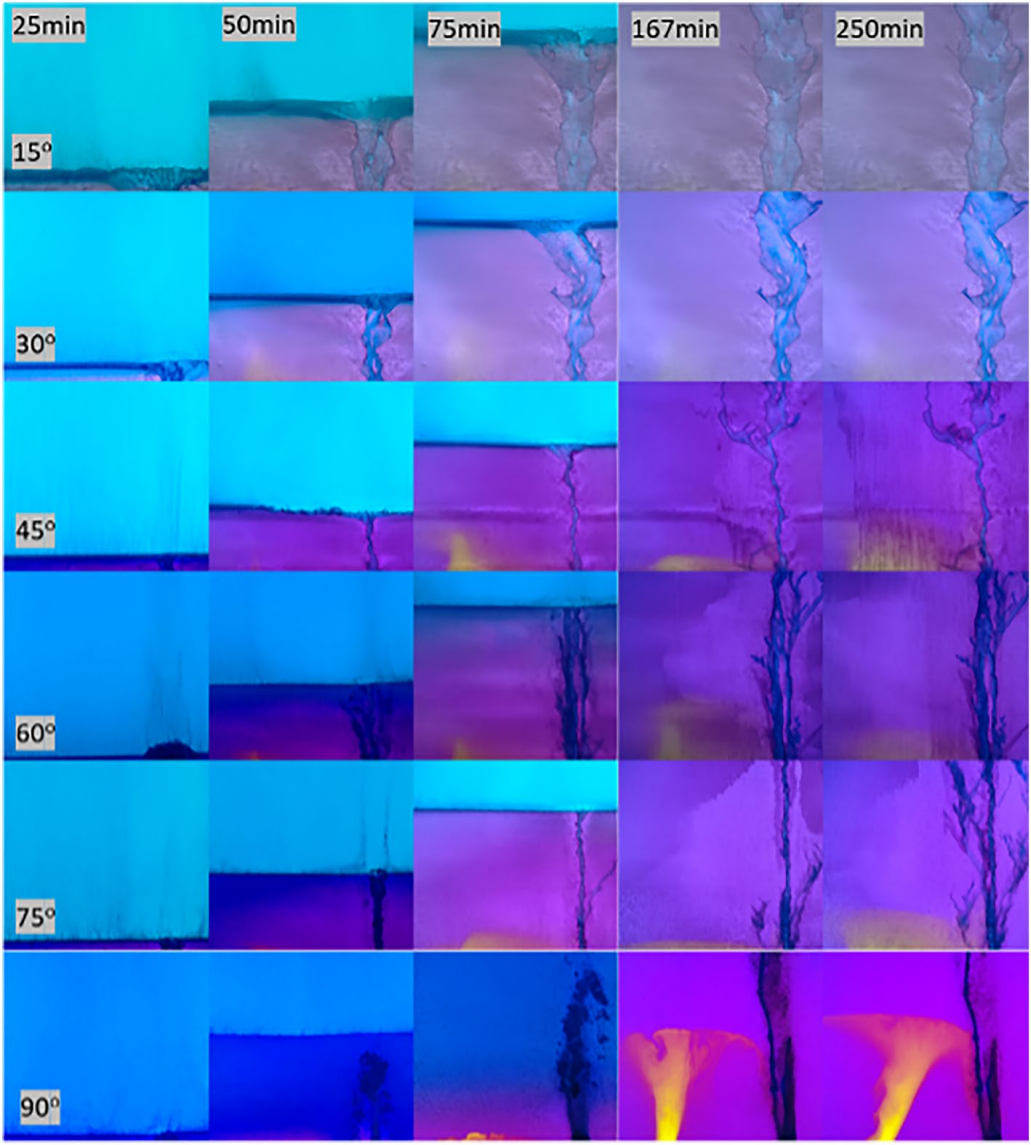
Development of precipitates in a fracture plane over time. The fracture orientation relative to gravity is given in the lower left hand corner of each row and the time at the top of each column. Colors are used to identify the fluid components based on pH (yellow–Solution 3, blue–Solution 4, purple–mix of solutions 3 & 4) and whitened regions contain calcite precipitates. Note: Solution 3 is pumped in on the left and Solution 4 from the right port. (For details on the experimental set-up see the Supplemental Information section 2 and for movies of the precipitation formation see Supplement movies M1 - M6).

Figure 7 provides a comparison of the $\hbox {CaCO}_3$ precipitate distribution for fractures with inclinations of 0$^\circ$ (fracture plane perpendicular to gravity) and 90$^\circ$ (fracture plane parallel to gravity). In the horizontal fracture (0$^\circ$ Fig. 7a), $\hbox {CaCO}_3$ precipitates are observed across the entire fracture plane because mixing occurs along the interface between the two fluids, i.e., the interface is essentially horizontal as the less dense fluid rides atop the denser fluid (Fig. 3). The precipitate distribution differs in the vertical fracture (90$^\circ$ Fig. 7b). A narrow runlet of calcium carbonate precipitates is observed above the port with the less dense fluid ($\hbox {Na}_2$ $\hbox {CO}_3$ Port in Fig. 7b). To observe how the fluid components mix during reactive miscible flow in inclined fractures, experiments were also performed with pH dye indicators added to the solutions (Table S2). Solution 3 was dyed with bromocresol purple, which is initially yellow (pH < 5.2). The less dense Solution 4 was dyed with bromocresol green, which is initially blue (pH > 5.4). When the two solutions mixed in a fracture, the pH increased and exceeded 6.8, causing the mixed fluids to turn purple. Figure 8 shows digital images of the fracture for 25, 50, 75, 167 and 250 minutes after the initiation of simultaneous pumping of solutions 3 and 4. Just as for the non-reactive case (Fig. 1), the fracture was initially filled with less dense Solution 4 (blue in Fig. 8 at 25 minutes) for the reactive miscible fluids experiment. As the less dense solution 4 is displaced and mixed with the denser Solution 3, the color changes to purple.

The effect of fracture inclination angle on fluid mixing is manifested in two ways: (1) the spatial distribution of calcium carbonate precipitates, and (2) the thickness of the precipitates. For all fracture inclinations, mixing between the two fluids occurred initially across the entire fracture plane because the fracture was initially saturated with the light solution (Solution 4). When both solutions are simultaneously pumped into the fracture, the denser Solution 3 sank and then displaced the lighter Solution 4 forming a uniform front, i.e., a horizontal line that spans the fracture plane (Fig. 8 for times 25-75 minutes and see movies SM1 - SM6 in Supplemental Information to view initial precipitate front). Unmixed dense fluid is observed near the inlet port for times $> 75$ mins and inclination angles $>30^\circ$ Once the denser solution reached the outlet (time >75 min), the less dense solution replenished the front leading to continual formation of precipitates along the horizontal front. However, depending on the fracture inclination, the precipitates either settled (i.e. rained down) from the horizontal front and accumulated around the inlet of the fracture, or deposited over the entire fracture plane. The sedimentation of the precipitates in regions near the inlet occurred for fracture inclinations of 45$^\circ$ to 90$^\circ$. While precipitates continually rained down from the front for fractures inclined at 90$^\circ$, a critical mass of precipitates was required for inclination of 45$^\circ$ to 75$^\circ$ case. When a critical mass was reached, the precipitates slid down the inclined fracture plane and collected near the inlet of the fracture. This was not observed for fractures inclined at 15$^\circ$ or 30$^\circ$. This suggests that the coefficient of static friction for the precipitates is between tan (30$^\circ$) and tan (45$^\circ$), though one must also account for viscous drag forces from the flowing solutions.

For fracture inclinations from 45$^\circ$–90$^\circ$, yellow appears in the image at times > 75 minutes, indicating that a high concentration of Solution 3 that is not interacting with the less dense Solution 4. As in the non-reactive case, the less dense fluid is essentially confined to a narrow runlet (blue path on right of images) as the denser fluid filled the fracture. As a result, after the initial displacement of the less dense fluid, precipitate formation was restricted to a narrow path along the less dense fluid runlet (Fig. 8) for high inclination angles. The precipitation along the edges of the runlet was sufficient to block flow in the aperture at these locations thus inhibiting mixing and the formation of additional precipitates.

**Figure 9:**
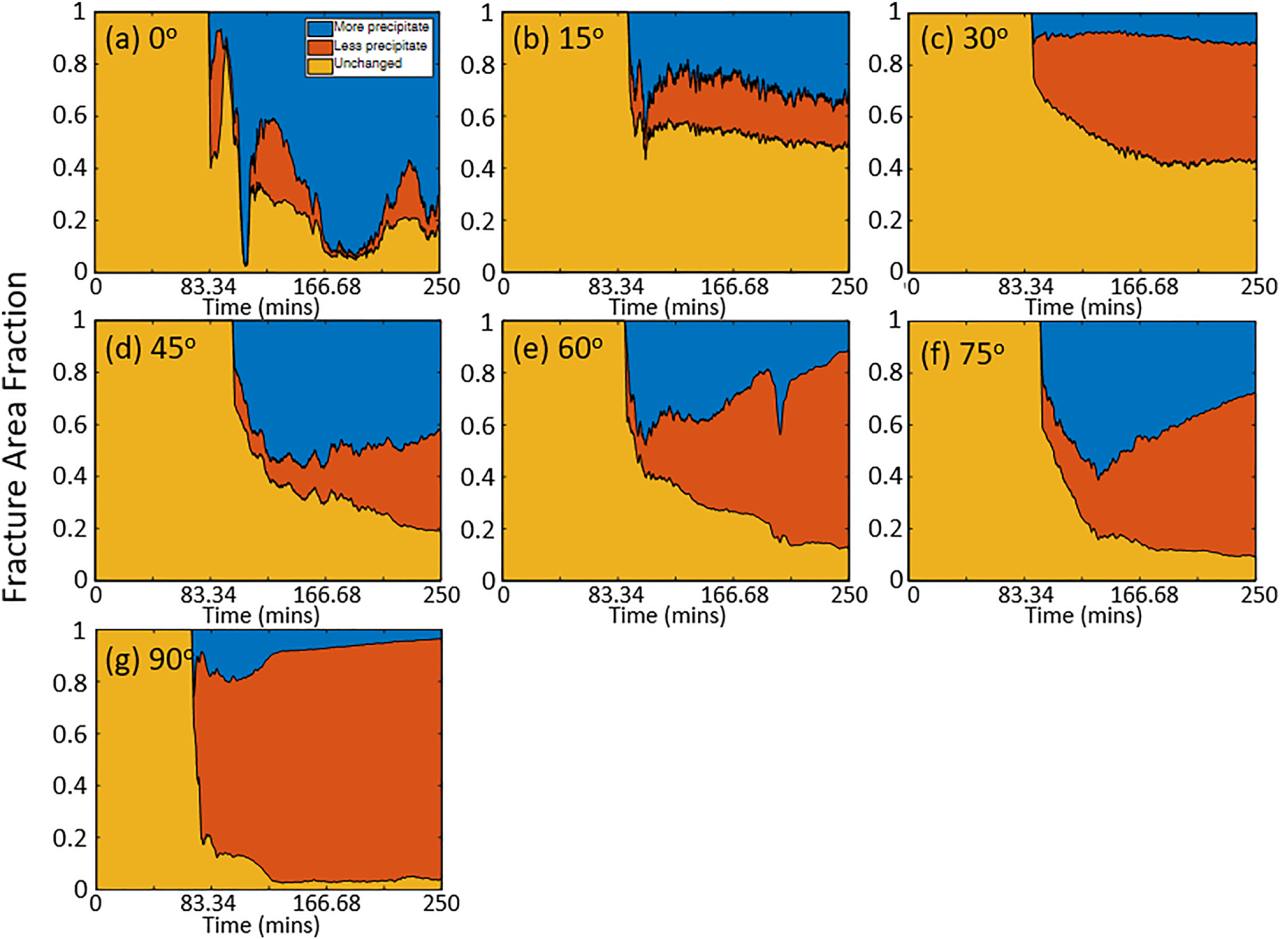
The fraction of the fracture plane area covered with more (blue) or less precipiates (red) or unchanged (yellow) relative to amount of precipitates after the reaction front reached the outlet for fracture inclination angles of (**a**) $0^\circ$, (**b**) $15^\circ$, (**c**) $30^\circ$, (**d**) $45^\circ$, (**e**) $60^\circ$, (**f**) $75^\circ$ and (**g**) $90^\circ$.

The change in precipitate extent across the fracture plane is shown in Fig. 9. The reference image was defined as the image taken after the reaction front reached the outlet. The reference image was subtracted from all images to quantify the change in the amount and spatial distribution of precipitates. The pixel intensity value decreases when precipitates are present because precipitation blocks light that is transmitted through the fracture. If the light intensity decreased, that pixel in the image was defined as having more precipitates relative to the reference image. Conversely, if the light intensity increased, the pixel was defined as having less precipitates. If the intensity did not change, a pixel was labeled as unchanged. Figure 9 shows the area fraction of precipitates across the fracture plane as a function time (where t = 0 is the reference frame) for different inclination angles. The area fraction was determined by counting the pixels that represent a certain condition (i.e. unchanged, more or less precipitates) divided by the total number of pixels that define the fracture plane. Regions are labeled as either more precipitates (blue), fewer precipitates (orange), or an equal amount of precipitates (yellow) relative to reference image. Regions with less precipitates increased with increasing inclination angle. At 90$^\circ$, most of the fracture plane (93%) lost precipitates compared to just after the passage of the reaction front (83 min). An inclination of 60$^\circ$ also exhibited a significant (73.94%) loss of precipitates across the fracture plane from sedimentation, while the 75$^\circ$ fracture showed 62.65% loss of precipitates. The loss of precipitates was significantly lower for 45$^\circ$ (36.99%), 30$^\circ$ (51.07%, 15$^\circ$ (16.72%) and 0$^\circ$ (14.18%). The loss of precipitates is mainly driven by sedimentation especially for large inclination angles. For small inclination angles, for example 15$^\circ$, the change is relatively small with the areas with more precipitate increasing slightly over time and the areas with less and equal precipitate showing a slight decrease. $\theta = 0^\circ$ is quite different from the other angles in that precipitate lost mainly arises from transport of precipitates out of the fracture. Most of the area (66.19%) has more precipitate compared to the reference image. Simulation results from (Sahu et al., 2009) showed that a gravity tongue of lighter fluid would form in a horizontal fracture, which aids in the potential mixing of two solutions over the entire fracture plane and thus the formation of more precipitates.

To summarize, in a uniform aperture fracture: (1) Unimpeded sedimentation occurs at an inclination angle of 90$^\circ$; (2) When the inclination angle $\theta > 30^\circ$, precipitates slide down to the bottom of the fracture; (3) When the angle is $45^o< \theta < 90^\circ$, precipitates collect near the inlet; (4) When $\theta < 30^\circ$, precipitates achieve almost complete coverage of the fracture plane. (The evolution of the precipitate distribution can be viewed in movies SM1-SM6 that are part of the Supplemental Information.)

## Homogeneous versus heterogeneous precipitation

**Figure 10:**
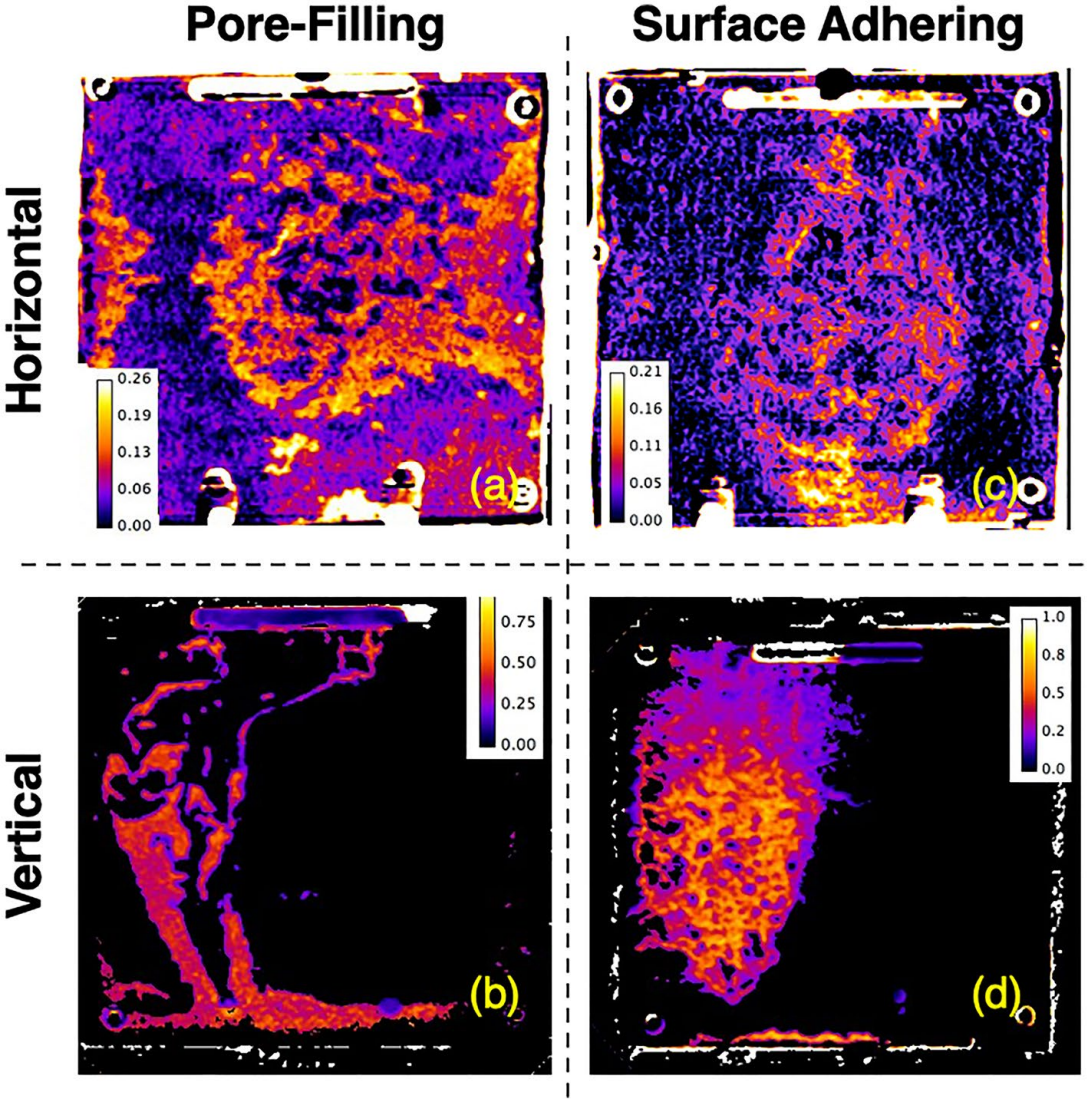
Comparison of (**a**, **b**) homogeneous or pore-filling and (**c**, **d**) heterogeneous or surface adhering precipitation in a rough walled fracture for fracture inclinations of (**a**, **c**) $0^\circ$ (horizontal) and (**b**, **d**) $90^\circ$ (vertical). The color scales represent precipitate thickness in millimeters.

In subsurface geological $CO_2$ sequestration induced precipitation may range from homogeneous to heterogeneous precipitates. Whether homogeneous (pore-filling) or heterogeneous (surface adhering) precipitation will occur in subsurface $CO_2$ sequestration will depend on the fluids, temperature conditions and rock mineralogy. Experiments were performed to determine if precipitate distribution differs whether homogeneous or heterogeneous precipitates are formed in a rough-walled fracture by the mixing of 2 miscible fluids for fracture inclinations of $0^\circ$ and $90^\circ$. A rough-walled fracture was created from poly-urethane casts of an induced fracture in Austin chalk (see Supplemental Information section 3 for fabrication details). The reaction for homogeneous precipitation is given by equation 1. Heterogeneous precipitates were created (Fig. 6b) using Solution 5, a 1 mol/L concentration calcium chloride ($CaCl_2$) aqueous solution, and Solution 6 was a 0.6 mol/L concentration sodium bicarbonate ($NaHCO_3$) aqueous solution (see Supplemental Information Table S3). The reaction of these two solutions results in

2 $$\begin{aligned}{} & {} {Ca^{2+} + H_2O \leftrightarrow Ca(OH)^+ + H^+} \end{aligned}$$

3 $$\begin{aligned}{} & {} {H^+ + HCO_3^- \rightarrow H_2O + CO_2(g)} \end{aligned}$$

4 $$\begin{aligned}{} & {} {Ca^{2+} + 2HCO_3^- \rightarrow CaCO_3(s) + H_2CO_3(aq)} \end{aligned}$$

that lead to calcium carbonate ($CaCO_3$) surface adhering precipitates and the production of carbon dioxide ($CO_2$) gas. As in the non-precipitation experiments, the fracture was initially filled with the less dense Solution 6 and then both solutions were simultaneously pumped into the fracture at a constant flow rate (0.17 ml/min) for 5 hours. Digital images were acquired every 5 seconds. Note, for these experiments and the results shown in Fig. 10, the less dense fluid was introduced through the left port and the higher density fluid through the right port which differs from non-reactive miscible fluid experiments shown in Figs. 1, 2, 3, and 5 and homogeneous precipitation in the smooth-walled fracture shown in Figs. 7 and 8. Post-experiment the fractures were X-ray CT scanned to determine the distribution and thickness of the calcium carbonate precipitate.

Figure 10 compares the precipitate distribution for the pore-filling and surface adhering calcium carbonate. For a fracture inclination of $0^\circ$, both the homogeneous (Fig. 10a) and heterogeneous (Fig. 10c) precipitates are observed across the entire fracture plane. The variation in thickness depends on the fracture aperture distribution and the waviness or rugosity of the fracture surface. The aperture will control the flow rates through the fracture while the waviness, especially for horizontal fractures ($0^\circ$), affects gravity segregation in the fracture that enables the less dense fluid to ride atop the denser fluid. For $90^\circ$, both the homogeneous (Fig. 10b) and the heterogeneous (Fig. 10d) precipitates are restricted to only a fraction of the fracture plane. However, the width of the precipitates is wider than that observed in homogeneous precipitation for the planar fracture Fig. 8. In a numerical study, Cao et al.^21^ showed that the shape, width and stability of a runlet in vertical fracture is affected by the variability in fracture aperture because of the variation in flow rates across the fracture plane. Gravity driven controls on calcium carbonate precipitates occur for both homogeneous and heterogeneous precipitation but the width of precipitate distribution in vertical fractures will be affected by surface roughness and in turn the aperture distribution.

## Discussion

Society’s ability to meet many of the current and future energy challenges (e.g., $CO_2$ sequestration) depends on our ability to predict how fluids behave and move through fractured rock in the subsurface where the fluids can potentially interact with the rock and naturally occurring fluids that reside there. In this study, a simplified chemistry with well-understood behavior and inert fracture walls were used for direct visualization of carbonate precipitation and deposition in fractures with different orientations. Our experimental observations and numerical simulations demonstrate that fluid mixing in a fracture is strongly dependent on the fracture orientation when there is a density contrast between two fluids, in this case of roughly 7%. The fracture plane orientation relative to gravity controls the segregation of fluids by density, leads to fluid confinement by the denser fluid (Fig. 1) which in turn affects the size and distribution of mixing lines or interfaces (Fig. 1) and the distribution of precipitates (Fig. 7). When a fracture plane is parallel to the direction of gravity, mixing and precipitate formation is strongly restricted to a narrow runlet. Though in a purely laminar flow condition, vortices are formed that control the stability of the narrow runlet (Fig. 5). Vortices occur because of gravity-driven segregation and confinement that results in a velocity difference between the two fluids. As the fracture inclination decreases, the runlet area increases and at $0^\circ$ (horizontal fracture) results in complete coverage of the fracture plane with precipitates. Gravity-driven effects were observed to occur for both homogeneous and heterogeneous precipitate distributions (Fig. 10b, d). For carbon mineralization in mafic and ultramafic rocks, injected $CO_2$ saturated water is denser and fully miscible with the ambient groundwater. Carbon mineralization relies on the dissolution of cations which then react with $CO_2$. In this scenario, both homogeneous and heterogeneous reactions are relevant. As long as relative fluid density is changing from mixing, precipitation and/or geochemical processes, there is the potential for gravity-driven chemical dynamics to control precipitate distributions.

It is important to keep in mind that in a subsurface $CO_2$ sequestration reservoir, the fluids and the chemistry are dramatically more complicated^22–24^. For fractured saline aquifers, the density contrast for $CO_2$ sequestration between liquid $CO_2$ (1101 $kg/m^3$) and water (1025 $kg/m^3$) is roughly 7%, similar to the density contrast used in our experiments. However, the density contrast between supercritical $CO_2$ and some subsurface brines can be as high as 50-70%^2^ which could potentially lead to stronger fluid segregation and confinement. Sequestration fluids, although significantly affected by the presence of dissolved $CO_2$, are also affected by temperature gradients, rock composition along the fracture surfaces, and brine chemistry. These factors will certainly affect precipitation. For example, reactions with the dissolved constituents from the rock (essentially a chemical soup) are likely to occur over numerous supersaturation thresholds instead of the single supersaturation as that applied in our experiments. However, despite the complexities of water-gas-rock interaction in a basin or tectonically active area, it is important to note that observed associated vein mineralogy is far less complicated - i.e., typically dominated by either carbonate or quartz (e.g.,^25–27^). For example, veins in mudrock observed in some parts of the Wolfcamp Formation consist of early dolomite followed by calcite and finally very minor quartz^28^.

Other fluid, rock and fracture properties also affect fluid mixing, mineral formation, and fluid and mineral distributions. A study based on the CarbFix geology determined that the type of minerals formed depended on the pH of the fluids with siderite forming for pH <5 and Mg-Fe- and Ca-Mg-Fe-carbonates for pH >5. For higher pH values, there is the potential to form Al- and Fe-hydroxides, chalcedony, and zeolites and smectites^24^. As these reactions occur along fracture flow paths, the pH and other fluid properties are likely to evolve over time and distance. Diffusivity of the fluids also affects mixing and alters the density contrast of fluids over time. High values of diffusivity will most likely lead to an increase in the runlet width or possibly inhibit runlet formation if the diffusion is rapid relative to the flow rate. The injection rate of the fluids will affect the stability of the runlet because it controls the shape and movement of vortices. Fracture and rock properties such as fracture aperture variability should be considered in future studies because the structural heterogeneity will affect runlet formation and the amount of fluid stratification within each aperture. In nature, fracture surfaces are rough and vary in mineralogy that result in aperture variability, and in turn can lead to preferential flow paths and stagnation zones, both of which are known to significantly affect fluid flow, mixing and transport as observed Fig. 10.

Our findings also suggest that the extent of carbon mineralization in natural fracture networks in rock will be affected by the orientation of the fractures within the network and the density contrast between any naturally occurring fluids and the engineered injected fluid. At the CarbFix site, columnar jointing^29^ is observed that formed during the cooling of lava. In general, fractures in a reservoir and their orientations are the result of many processes, including tectonic, thermal, chemical, and rock properties, and are not likely to be known a priori. Fractures are often formed over time in response to different stimuli (cooling, heating, tectonics, dissolution, and precipitation affected by fluid flow) that have different time constants and may occur simultaneously or sequentially. In the simplest conceptualization, the imbalance of principal stresses would result in mostly vertical fractures at greater depth, and horizontal fractures at shallow depths^30^. Site selection would require knowledge of pre-existing fractures or fracture networks and the potential changes in fluid properties during injection and chemical reactions to maximize $CO_2$ trapping through in-situ mineralization in fractures. This raises the question that if horizontal fractures are present, will they preferentially seal? Sealed horizontal veins, referred to as “beef” veins, are commonly observed particularly in sedimentary basins^27^ and often serve as bedding parallel seals in shale. The precipitation observed in our experiments requires that at least one fluid is flowing and in contact with a second fluid. As a fracture becomes sealed, the flow would be reduced because the flow resistance would increase from sealing, aperture-reduction and/or plugging from precipitates. A fully sealed fracture would no longer support flow nor enable the mixing mechanisms observed in this study unless volume expansion commonly associated with carbonate and associated secondary mineral precipitation-induced cracking^31^ occurred that reconnected the flow paths of both fluids.

In summary, our tests demonstrate some of the fundamental physical and chemical processes that can affect the extent of fluid mixing and the distribution of mineral precipitates that result from homogeneous or heterogeneous precipitation in a fracture. The potential for gravity-driven chemical dynamics should be considered when selecting and designing future subsurface sites for mineral trapping of $CO_2$. While studies have examined the chemical reactions that are inferred to have occurred in the subsurface at CarbFix^23^, the effect of these reactions on fluid properties, gravity-driven mixing and extent and transport of precipitates as they occur inside fractures has largely been ignored. Simulations of reservoir flow is often performed at the continuum scale, a scale that cannot account for the details of the fracture geometry ( i.e , surface roughness, aperture distribution, void shape, contact area, etc.), variation in fracture surface mineralogy, evolution of fluid properties nor gravity-driven chemical reactions, all occurring within a fracture network. These processes need to be address in light that the chemistry will be extremely more complicated than that used in our experiments to demonstrate the existence and potential for gravity-driven chemical dynamics at a $CO_2$ subsurface site.

## Methods

**Sample:** Uniform aperture fracture samples were created from two flat transparent polycarbonate (PC) plates to enable optical imaging of the injected fluids. The plates measured 100 mm x 100 mm x 12.7 mm (Figure S1 Supplemental Information).

### Chemical formulations

Experiments with non-reactive and reactive fluids were performed to understand how density contrast alone affects the mixing of two fluids and precipitation of calcium carbonate in inclined fractures. The experimental solutions used for the non-reactive experiments are listed in Table S2 in the Supplemental Information.

### Fluid pumping

Two Harvard Apparatus syringe pumps were used to introduce, simultaneously, two solutions into a fracture. The pumps were connected to the two inlet ports on the sample with 1/8-inch diameter PFA tubing and 1/16 inch MNPT Swagelok fittings. One 200 mL syringe contained Solution 1 and the other syringe contained Solution 2 (see Tables S1 & S2). The fracture was initially saturated with the less dense Solution 2. Then the two solutions were simultaneously pumped into the fracture at the selected constant flow rate (0.17 ml/min) for 5 hours to enable mixing and the formation of mineral precipitates. After 5 hours, the pumps were turned off.

### X-ray imaging

X-ray computed tomographic (CT) imaging was performed to image 3-D spatial distribution of precipitates during the experiment. Scanning was performed at Lawrence Berkeley National Lab using a modified General Electric Lightspeed 16 slice medical X-ray computed tomography (CT) system at 80 and 120 kV, 200 mA. Reconstructed images were processed using open-source ImageJ software and associated plug- in packages.Scans were sequentially performed at 120kV and 80 kV. Pre-experiment tests were performed showing that the calcite is much less transparent to the 80kV energy. All the other materials behaved similarly at the 2 energies. In taking the difference between the two scans, the calcite-rich regions are accentuated.

### Imaging & image analysis

A custom-built digital optical imaging system was used to record images of the fracture prior to, during and after flowing both solutions into the fracture. The system consisted of a Spy camera for a Raspberry Pi with a native resolution of 5 Megapixels yielding 2592 x 1944-pixel images. The camera was connected to a Raspberry Pi Model B+ with 512MB RAM. The images were recorded every 5 seconds and stored as jpeg files directly to a 128 GB flash drive. Description of imaging calibration and lighting is given in the Supplemental Information section 5.

### Numerical modeling

An opensource CFD software OpenFOAM (OpenFOAM: The Open Source CFD Toolbox , 2014) was used to simulate gravity-driven flow and transport of miscible fluids of different densities in a vertical fracture. We developed an OpenFOAM solver by coupling a flow solver (buoyantBoussinesqPimpleFoam) and an advection-diffusion solver (scalarTransportFoam). Additional details are given in the Supplemental Information in section 6. Fluid properties used for the simulation are given in Table S5.

## Supplementary Information

Supplementary Information 1.

Supplementary Information 2.

Supplementary Information 3.

Supplementary Information 4.

Supplementary Information 5.

Supplementary Information 6.

Supplementary Information 7.

Supplementary Information 8.

Supplementary Information 9.

## Data Availability

The data are available in the Purdue University Research Repository (PURR) Publications repository, under the Xu, Z., Cao, H., Yoon, S., Kang, P., Jun, Y., Kneafsey, T., Sheets, J., Cole, D., Pyrak-Nolte, L. (2023). Data for Gravity Driven Chemical Dynamics in a Single Fracture. Purdue University Research Repository. 10.4231/657J-V831 and can be accessed with through the following link: https://purr.lib.purdue.edu/registry/dataset/10_4231_657J_V831.
