# Supplementary material for: Gravity-driven controls on fluid and carbonate precipitation distributions in fractures
Source: Sci Rep. 2023 Jun 9;13:9400. doi: 10.1038/s41598-023-36406-8 (PMC10256864; doi:10.1038/s41598-023-36406-8)
Supplement: Supplementary file 1 — Supplementary Information 1. [file 41598_2023_36406_MOESM1_ESM.pdf]

Supplementary Information: Gravity-Driven Controls on Fluid and Carbonate Precipitation Distributions in Fractures

Zhenyu Xu[1+], Hongfan Cao[2+], Seonkyoo Yoon[2], Peter K. Kang[2+], Young-Shin Jun[3], Timothy Kneafsey[4], Julia M. Sheets[5], David Cole[5], and Laura J. Pyrak-Nolte[1,6,7+*]

[1] *Purdue University, Department of Physics & Astronomy, West Lafayette, Indiana, 47907, USA*
[2] *University of Minnesota, Department of Earth and Environmental Sciences, Twin Cities, Minnesota, 55455,USA*
[3] *Washington University in St. Louis, Department of Energy, Environmental and Chemical Engineering, St Louis, Missouri,63130,USA*
[4] *Lawrence Berkeley National Laboratory, Earth & Environmental Sciences, Berkeley, California, 94720,USA*
[5] *The Ohio State University, School of Earth Sciences, Columbus, Ohio, 43210,USA*
[6] *Purdue University, Department of Earth, Atmospheric & Planetary Sciences, West Lafayette, Indiana, 47907, USA*
[7] *Purdue University, Lyles School of Civil Engineering, West Lafayette, Indiana, 47907, USA*
[*] *Corresponding author's email: ljpn@purdue.edu*
[+] *These authors contributed equally to this work*

May 6, 2023

1 Introduction

Here, additional information is provided on the experimental and computational approaches used for this study. This includes sample preparation, fluid properties, digital image calibration, and the equations related to the computational simulations. We also include some movies of the reactive and nonreactive experiments.

2 Sample Preparation - Uniform Aperture Fractures

Synthetic fractures were created from polycarbonate (PC) plates that measured 100 mm x 100 mm x 12.7 mm (Fig S1 supplemental information). Two inlet ports were drilled 38 mm apart into upper plate of the sample and an outlet slot was cut into the lower plate that also contained a hole to connect a tube to the outlet (Figure S1a). The edges of two fracture surfaces were separated by PTFE (Polytetrafluoroethylene) sheets to create uniform apertures of 2 mm. The two fracture surfaces were screwed together and sealed with PDMS (Polydimethylsiloxane) and silicone rubber sealant made by DAP, Inc. The sample was mounted on an easel to control the inclination angle of the fracture (Figure SS1b). A Digi-Pas DWL-80E Digital Leveler to measure the angle of inclination. A RaspberryPi spy camera was mounted on a rod fixed attached to the easel to ensure that the distance between the camera and the fracture plane was the same for all fracture inclinations.

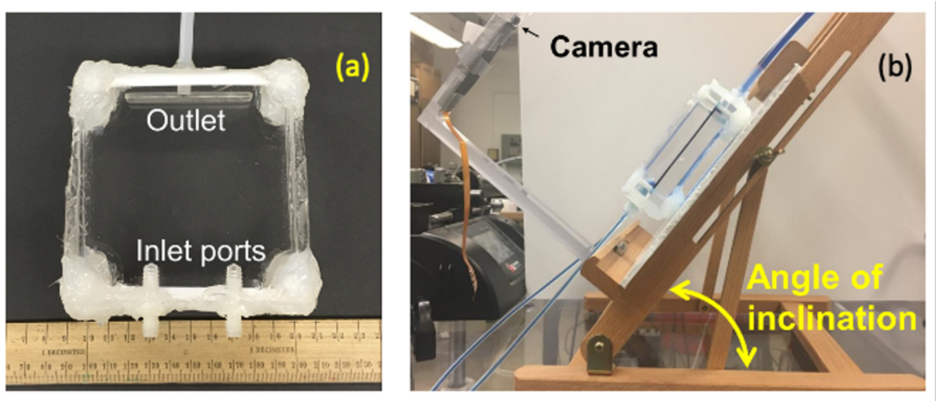

Figure S1: Digital image of (a) a sealed fracture sample showing the location of the inlet and outlet; and (b) the fracture sample mounted on an easel to control inclination of the fracture plane.

3 Sample Preparation - Variable Aperture Fractures

A variable aperture fracture was created through a casting process. A sample of Austin chalked with a single fracture induced using a method similar to Brazil[1] or split cylinder testing was used to create a mold. After building a chamber around each half of the fracture, Alumilite's High Strength 2 silicone rubber was poured into the space between the wall and the Austin chalk sample. After 24 hours of curing, the Austin chalk was removed from the rubber thereby creating a mold of the fracture surface. Before casting with urethane, the rubber mold was preheated in the oven at 66^oC for 30 minutes. Alumilite Water Clear casting resin A and resin B were mixed with equal parts by weight and poured into the rubber mold. The mold with the resin was then transferred to a pressure tank, and placed under 20 psi (138 kPa) air pressure. The resin-filled mold was held under pressure for 24 hours while it cured. The cast sample measured 100 mm $\times$ 100 mm $\times$ 78 mm (Figure S2) with two inlet ports located 38 mm apart into the upper half of the fracture sample and day-lighted on the upper fracture surface . The outlet port consisted of a slot cut into the lower fracture surface with a drilled hole for outlet tubing. The two fracture surfaces were placed in contact, screwed together at the corners, and sealed with DAP silicone rubber sealant to prevent any leaks. Figure S3 is a contour map and histogram of the fracture aperture distribution based on laser profilometry data (a laser LK-G152, from Keyence Corporation, measurable height range of the laser was +/-9.969 mm, with an accuracy of 0.5 μm, with 0.25 mm stepsize). The asperity height of each surface was measured. Cross-correlation was used to align the measurements from the each fracture surface prior to determining the aperture. Only the central portion of the fracture between the inlet and outlet is shown in Figure S3.

4 Fluid Properties

Experiments were performed using both nonreactive and reactive miscible fluids. The densities of Solution 1 and 2 (Table S1) were set to be the same as the Solutions 3 & 4 used in the precipitation experiments (Table S2) and for Solutions 5 & 6 (Table S3) to maintain the same density contrast between the injected fluids for all experiments. A small amount of pH indicators was added to make the solutions distinguishable. Bromocresol green was added to Solution 1. Bromocresol green is blue when pH is above 5.4, and changes to yellow at pH value below 3.8. In the reactive experiments, homogeneous mineral precipitation of calcium carbonate ($CaCO_3$) was induced in a

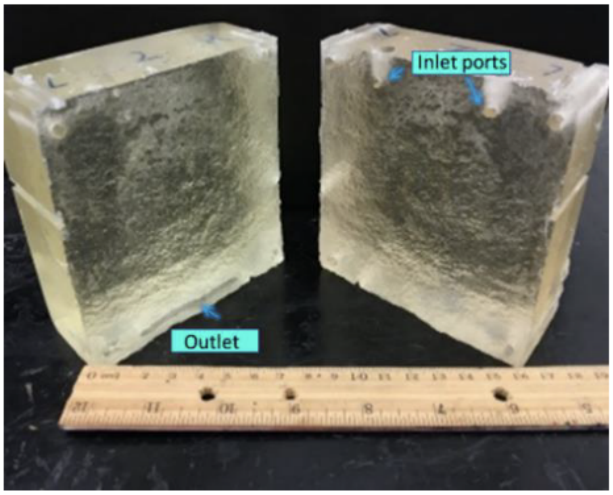

Figure S2: Digital image of the acrylic rough-walled fracture sample based on an induced fracture in Austin Chalk.

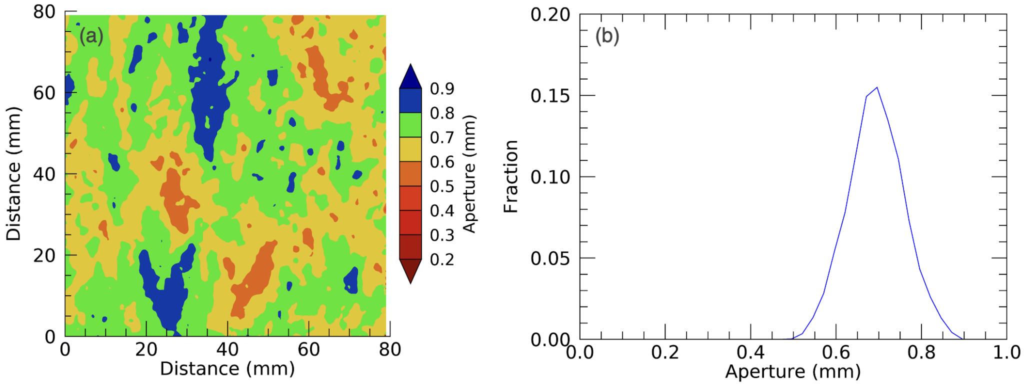

Figure S3: (a) Contour map and (b) histogram of the aperture distribution of the acrylic fracture sample.

fracture by mixing of Solutions 3 & 4 (Table S2) that resulted in the following reaction:

$$CaCl_2 + Na_2CO_3 \rightarrow CaCO_3(s) + 2NaCl \tag{1}$$

The $CaCO_3$ products form in solution (homogenous precipitates). Bromocresol purple was added to Solution 3 and bromocresol green was added to Solution 4. Bromocresol purple changes color from yellow at pH below 5.2 to purple for pH above 6.8. Solution 3 was initially yellow. When the solutions mixed, the pH increased and exceeded 6.8, causing the mixed fluids to turn purple in color. Solution 4 was initially blue as the pH was larger than 5.4. When Solutions 3 & 4 were mixed, the saturation index (Log(IAP/Ksp calcite) of the mixed solution was 5.16 where IAP is the ion activity product and Ksp is the solubility product of calcite. The solution pH was 11.

Solutions 5 & 6 (Table S3) were used to generate heterogeneous precipitates. The mixing of these two solutions results in:

$$Ca^{2+} + H_2O \leftrightarrow Ca(OH)^+ + H^+ \tag{2}$$

Table S1: Components of Solutions 1 & 2 for the miscible fluid experiments

	Component	Amount(g)	Density (kg/m^3)	Viscosity (Pa-s)
	Na_2CO_3	2.07		
Solution 1	NaCL	9	1111	1.20 x 10^{-3}
	Bromocresol Green	0.03		
	H_2O	100		
Solution 2	Na_2CO_3	3.18	1031.8	1.08 x 10^{-3}
	H_20	100		

Table S2: Components of Solutions 3 & 4 for homogeneous calcium carbonate precipitation experiments.

	Component	Molarity (mol/L)	Amount (g)	Density (kg/m^3)	Viscosity (Pa-s)
	$CaCl_2 * 2H_2O$		14.67		
Solution 3	H_2O	1.0	96.4	1111	1.23 x 10^{-3}
	Bromocresol Purple		0.03		
	Na_2CO_3		3.15		
Solution 4	H_2O	0.3	100	1031.8	1.08 x 10^{-3}
	Bromocresol Green		0.03		

$$H^+ + HCO_3^- \rightarrow H_2O + CO_2(g) \tag{3}$$

$$Ca^{2+} + 2HCO_3^- \rightarrow CaCO_3(s) + H_2CO_3(aq) \tag{4}$$

such that the excessive $CaCl_2$ leads to an acidic solution, and H^+, calcium carbonate ($CaCO_3$) surface adhering precipitates, and the production of carbon dioxide (CO_2) gas.

5 Calibration of Digital Imaging

An Air Force Test Chart (MIL-STD-150A, 1951 USAF resolution test chart) (Fig S4) was used to determine the pixel edge length (65.96 µm/pixel) in the RaspberryPi images. Calibration experiments were also performed with four solutions to aid in the determination of fluid concentrations from image analysis. The solutions used in the calibration consisted of concentrations of Solution 1 of 25%, 50%, 75% and 100% by weight (see Table S4) with Solution 2 (see Table S1). The Lambert-Beer law shows that there is a linear relationship between the intensity of light absorbed by a substance dissolved in a solvent and the concentration of the substance as well as the path length of the light through the solution. In the experiments, the uniform aperture fracture was

Table S3: Components of Solutions 5 & 6 for heterogeneous precipitation of calcium carbonate.

	Component	Molarity (mol/L)	Amount (g)	Density (kg/m^3)	Viscosity (Pa-s)
	$CaCl_2$*$2H_2O$		14.7		
Solution 5	H_2O	1.0	96.4	1111	1.23 x 10^{-3}
	$NaHCO_3$		5.04		
Solution 6	H_2O	0.6	100	1050	9.90 x 10^{-4}

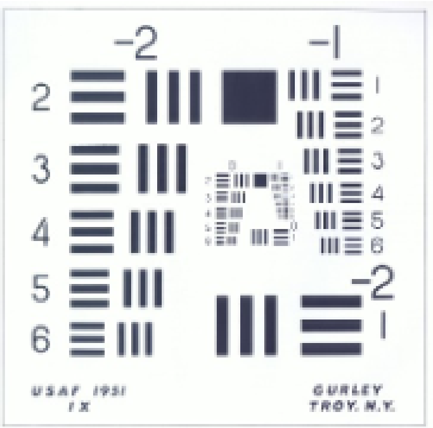

Figure S4: Air Force Test Chart used to calibrate the pixel edge length.

backlit with a Daylight Wafer 1 Light Box. A Digi-Sense Traceable light meter was used to ensure the illumination was the same among experiments. The recorded pixel intensity from the images is related to the light intensity after having passed through the solution-filled fracture. The light intensity is only affected by the concentration and thickness of the solution. The thickness of the solution depends on the inclination of the fracture plane (Figure 3 inset in the manuscript). As shown in Figure 3, the stratification of two fluids with a density contrast depends on the orientation of the fracture plane relative to gravity. There is no difference between a well-mixed and a poorly mixed fluid in terms of the transmitted-light intensity. For example, assume fluid A is composed of 50% colorless fluid 2 and 50% blue fluid 1 and are well-mixed such that fluid. A is in even blue color, and Fluid B is composed of one layer of 50% colorless fluid 2 on top of one layer of 50% blue fluid 1. The light intensity that passes through fluids A and B would be the same in these two situations as long as the path of the light that travels in the fluids is the same. When a reference is made to the concentration of mixed fluids, it can either be a well-mixed even color fluid, or possibly a layered fluid. This indicates that the ratio of different concentration fluids occupying the fracture can be obtained from experimental images, but not the true mixing state.

From a study on non-reactive miscible fluids, Tchelepi[2] defined a viscous-to-gravity ratio, R_{ν/g_z} to determine whether viscosity or buoyancy (gravity) will dominate during flow. R_{ν/g_z} is given by

$$R_{\nu/g_z} = \frac{\bar{v}\Delta\mu H}{k_z \Delta\rho g L} \tag{5}$$

where $\bar{v}$ is the mean Darcy velocity in the x direction, k_z is the characteristic permeability in z direction (2D case only has x and z directions), $\Delta\mu$ is the viscosity difference, $\Delta\rho$ is the density difference, H is the height and L is the length of the channel. When the mixing is completely dominated by gravity, the displacing fluid (with a smaller density) forms a "gravity tongue" covering top of the channel. If the mixing is totally dominated by viscosity, "fingers" appeared from the displacing fluid towards the displaced fluid. When using R_{ν/g_z} as a standard to judge the fluid behavior, when $R_{\nu/g_z} < 2$ gravity effects override viscosity effects in both 2D and 3D models. For our system, $\bar{v} \sim 0.13$ mm/s, $\mu \sim 0.001$ Pa-s, $H \sim 2.0e-03$ m, $L \sim 100.0e-03$ m, $\Delta\rho = 1110 - 1038$ kg/m3, $g = 9.8$ and $k = a^2/12 = 3.33e-07$. This yields $R_{\nu/g_z} \sim 0.8$ which puts our system in the gravity dominated regime and indicates that fluid segregation is likely to have occurred. Equation 5 was developed for two immiscible fluids. Additional research is needed to confirm the applicability of equation 5 to miscible fluids with a density contrast which is beyond the scope of the work.

Table S4: Solutions for calibrating Solution 1 concentration

Concentration w.r.t. Solution 1 (by weight)	Weight Solution 1 (g)	Weight Solution 2 (g)
25%	10	30
50%	20	20
75%	30	10
100%	40	0

6 Simulation Methods

The experimental results demonstrate that the mixing of two miscible fluids and precipitate distributions in a fracture are strongly affected by the fracture inclination angle when the two fluids have different densities because of gravity segregation. Runlet formation from the confinement of a less dense solution by higher density solution restricts the region of potential mixing or reactions to the perimeter of the runlet or essentially the surface area of the runlet. A key question is what maintains the geometry of the runlet and produces the observed instabilities that affect the shape of the runlet. To investigate the origin of the runlet and the cause of the runlet instability, we performed three-dimensional (3D) numerical simulations of non-reactive miscible fluids.

An opensource CFD software OpenFOAM ((OpenFOAM: The Open Source CFD Toolbox , 2014)) was used to simulate gravity-driven flow and transport of miscible fluids of different densities in a vertical fracture. We developed an OpenFOAM solver with a flow solver (buoyantBoussinesqPimpleFoam) and an advection-diffusion solver (scalarTransportFoam). The coupling is carried out by setting the fluid density term as a function of solute concentration. The solver captures the coupling between the fluid and solute transport: the fluid density variations determined by solute transport and mixing is captured by solving advection diffusion equation, and the fluid flow that honors concentration-dependent density is captured by solving the Navier-Stokes equations for incompressible flow with the Boussinesq approximation. Under the Boussinesq approximation, the governing equations for the mass and momentum conservations can written as:

$$\nabla \cdot \boldsymbol{u} = 0 \tag{6}$$

$$\rho_o(\frac{\delta \boldsymbol{u}}{\delta t} + \boldsymbol{u} \cdot \nabla \boldsymbol{u}) = -\nabla p + \rho \boldsymbol{g} + \nu \nabla^2 \boldsymbol{u} \tag{7}$$

where $\mathbf{u}$ is the velocity field, p is the pressure field, ρ is the fluid density, g is the gravitational acceleration, ρ_o is the reference density, and ν is the kinematic viscosity. Through the Boussinesq approximation, we consider the density variation in the gravitational term with the following equation:

$$\rho = \rho_o + \frac{\delta \rho}{\delta C}\left(C - C_o\right) \tag{8}$$

The solute transport in a fracture is described by advection-diffusion equation (ADE):

$$\frac{\delta C}{\delta t} + \nabla \cdot (\boldsymbol{uC}) - \nabla^2\left(D_{diff} C\right) = 0 \tag{9}$$

where C is the solute concentration, and D_{diff} is the diffusion coefficient. The numerical solver solves the flow and transport equations sequentially. The simulation setup is similar to the experiment with a vertical flow cell and the fluid properties listed in Table S5. The size of the domain

Table S5: Parameters for fluids used in simulations

	With Density Contrast		Without Density Contrast	
	Fluid 1	Fluid 2	Fluid 1	Fluid 2
Density (kg/m^3)	1111	1031.8	1031.8	1031.8
Dynamic Viscosity (Pa s)	1.20×10^{-3}	1.11×10^{-3}	1.20×10^{-3}	1.11×10^{-3}
Injection Rate (ml/min)	0.17		0.17	
Diffusion Coefficient (m^2/s)	10^{-9}		10^{-9}	

is 100 mm ×100 mm with a uniform fracture aperture of 2 mm. The entire domain is discretized into 400 × 400 × 16 cells, which gives the cell size of 0.25 mm × 0.25 mm ×0.125 mm. In the simulation, we set two rectangle inlet ports that are 38 mm apart at the bottom boundary of the fracture, with an inlet port with dimensions of 1.5 mm × 3 mm. A rectangle outlet port of with dimensions 1 mm × 60 mm is placed at the upper boundary of the fracture. We assign no slip boundary condition to all solid walls. The fracture domain is initially filled with the lighter fluid (same as the experimental condition). Then, the denser fluid is injected from the left inlet at a constant rate of 0.17 ml/min, and the lighter fluid is injected from the right inlet at the same rate as the denser fluid.

7 Media Uploaded Separately

Table S6 list the filenames of videos that show the evolution of the fluid mixing and the precipitate distribution across the fracture plane.

Table S6: Videos of Mineral Precipitation Experiments

File Name	Fracture Inclination Angle
SM1_Reactive_theta_0.avi	0°
SM3_Reactive_theta_30.avi	30°
SM3_Reactive_theta_45.avi	45°
SM4_Reactive_theta_60.avi	60°
SM5_Reactive_theta_75.avi	75°
SM6_Reactive_theta_90.avi	90°
SM7_Nonreactive_theta_0.avi	0°
SM8_Nonreactive_theta_90.avi	90°

The videos are provided to show the precipitate development in between the still frames shown in Figure 8 in the manuscript and the runlet development for an inclination of 90° shown in Figure 1 in the manuscript. Note the videos were made from still images recorded every 5 seconds for over the 5 hour period of each experiment experiment. The time of the videos is significantly shorter than the actual experiment. The port with the denser fluid is toward the lower left in the videos and the less dense fluid port is toward the lower right. Colors in the videos represent the different fluid components based on pH dye indicators with yellow - Solution 3, blue - Solution 4, purple - mix of solutions 3 & 4, and whitened regions contain calcite precipitates.

8 References

1. Jaeger, J.C, N.G.W. Cook, and R.W. Zimmerman, 2007, Fundamentals of Rock Mechanics: Blackwell Publishing

2. Tchelepi, H.A., Viscous Fingering, Gravity Segregation and Permeability Heterogeneity in Two Dimensional and Three Dimensional Flows, in Department of Petroleum Engineering. 1994, Stanford University.
